# Exhaust Emissions from Gasoline Vehicles with Different Fuel Detergency and the Prediction Model Using Deep Learning

**DOI:** 10.3390/s23177655

**Published:** 2023-09-04

**Authors:** Rongshuo Zhang, Hongfei Chen, Peiyuan Xie, Lei Zu, Yangbing Wei, Menglei Wang, Yunjing Wang, Rencheng Zhu

**Affiliations:** 1 School of Ecology and Environment, Zhengzhou University, Zhengzhou 450001, China; zhangrs4777@163.com (R.Z.); chf1210@gs.zzu.edu.cn (H.C.); xpy2022266@gs.zzu.edu (P.X.); wyb0110@gs.zzu.edu.cn (Y.W.); 202010107793@mail.scut.edu.cn (M.W.); 2 State Environmental Protection Key Laboratory of Vehicle Emission Control and Simulation, Chinese Research Academy of Environmental Sciences, Beijing 100012, China; zulei@vecc.org.cn (L.Z.); wangyj@ceaes.org.cn (Y.W.); 3 Research Centre of Engineering and Technology for Synergetic Control of Environmental Pollution and Carbon Emissions of Henan Province, Zhengzhou University, Zhengzhou 450001, China

**Keywords:** vehicle emission model, gasoline detergency, deep learning, on-road emissions, portable emission measurement system (PEMS)

## Abstract

Enhancing gasoline detergency is pivotal for enhancing fuel efficiency and mitigating exhaust emissions in gasoline vehicles. This study investigated gasoline vehicle emission characteristics with different gasoline detergency, explored synergistic emission reduction potentials, and developed versatile emission prediction models. The results indicate that improved fuel detergency leads to a reduction of 5.1% in fuel consumption, along with decreases of 3.2% in total CO_2_, 55.4% in CO, and 15.4% in HC emissions. However, during low-speed driving, CO_2_ and CO emissions reductions are limited, and HC emissions worsen. A synergistic emission reduction was observed, particularly with CO exhibiting a pronounced reduction compared to HC. The developed deep-learning-based vehicle emission model for different gasoline detergency (DPVEM-DGD) enables accurate emission predictions under various fuel detergency conditions. The Pearson correlation coefficients (Pearson’s r) between predicted and measured values of CO_2_, CO, and HC emissions before and after adding detergency agents are 0.913 and 0.934, 0.895 and 0.915, and 0.931 and 0.969, respectively. The predictive performance improves due to reduced peak emissions resulting from improved fuel detergency. Elevated gasoline detergency not only reduces exhaust emissions but also facilitates more refined emission management to a certain extent.

## 1. Introduction

Vehicle exhaust emissions are a major contributor to carbon emissions, and this concern is further amplified by the annual increase in the number of vehicles. Moreover, carbon monoxide (CO) and hydrocarbons (HC) are the primary pollutants in the exhaust emissions from gasoline vehicles. These pollutants can cause a wide range of acute and chronic symptoms in patients [1]. Despite the rapid development of new-energy vehicles, achieving a fully electrified mass transit system within a few years remains challenging. In the short term, gasoline vehicles would remain the main on-road passenger transportation.

In order to mitigate carbon and pollutant emissions from road traffic, many new technologies were introduced in the new vehicle to reduce the carbon dioxide (CO_2_), CO, and HC emissions of gasoline vehicles at the source. The Plug-in Hybrid Electric technique was proven as a useful method to decrease the emissions, especially under the state of charge [2,3,4]. Zhu et al. (2022) and Fonseca et al. (2011) revealed that CO_2_ emission would be lower with the start–stop system on [5,6]. These new technologies are aimed at reducing the emissions from newly produced vehicles, using some similar techniques such as lightweight material [7] and hybrid electric technology [8]. It is not practical to introduce them into the existing huge stock of motor vehicles in use. Some other new methods proposed could be applied in all vehicles, such as eco-routing navigation [9] and shortening vehicle lifetimes [10]. However, there is still a long way to go before these ideas can be realized. The key to reducing on-road emissions from all fuel vehicles is achieving a coordinated and comprehensive approach to emissions reduction that is cost-effective.

Fuel quality is a critical factor of vehicle emissions, which can significantly affect the exhaust emissions. Upendra et al. illustrated a notable decrease in exhaust pollutants by employing bio-blended fuels. Specifically, an 80% pure diesel mixed with a 20% blend of first-, second-, and third-generation biodiesels exhibited a distinct reduction in smoke and particulate matter (PM) emissions [11]. Moreover, the blend of 80% diesel and 20% microalgae spirulina demonstrated a reduction of 13.7% in NOx emissions and 22.2% in smoke emissions [12]. Additionally, the incorporation of 20% plastic waste oil with 80% diesel fuel led to improved engine performance coupled with reduced exhaust emissions [13]. The adoption of aegle methyl ester biodiesel led to a modest elevation in CO_2_ and NOx emissions, offset by substantial reductions in PM and smoke emissions [14].

Vehicle gasoline detergency refers to the efficacy of fuel in preventing deposits within engine components such as the fuel line, intake valve, and combustion chamber [15]. It reflects both the tendency to form carbon deposits during usage and the potential to remove existing deposits [16]. The addition of gasoline detergent was regarded as an economical approach to enhance gasoline detergency, achieving higher fuel consumption efficiency and lower pollutant emission [17]. Jin et al. (2021) organized 14 popular detergents from the China market and found that 9 of them can significantly reduce CO emissions by 8.5–28.3%, and most of the test gasoline detergents could reduce the fuel consumption by 0.9–3.5% [18]. Zand et al. (2007) found that gasoline detergents can reduce CO and HC emissions from gasoline engine vehicle exhaust by removing engine carbon deposits [15]. Zhu et al. (2016) further supported this discovery, demonstrating a 6.8% reduction in engine intake valve deposits and a 13.6% decrease in CO emissions [19]. Chaurasiya et al. (2022) found that 5% hydrogen added in ton-butanol could lead the lower CO_2_, smoke, NOx, and PM emissions [20]. However, the actual efficacy of its emission reduction remains inadequately understood in practical driving scenarios. The aspect of emissions reduction merits more comprehensive attention and scrutiny in the scientific discourse.

Analyzing real-world emission characteristics is crucial for innovating emission reduction techniques and ensuring accurate emission regulation. Numerous existing studies have explored vehicle emission features. Wang et al. conducted tests on CO_2_ emissions from two gasoline vehicles under four distinct environmental temperatures. The results showed that the CO_2_ emission deteriorated under cold ambient temperatures because of the increased internal resistance [21]. Zhu et al. (2022) used a machine learning model to confirm that engine load percent and engine speed were the most dominant factors affecting vehicular carbon emissions based on PEMS data [22]. Gallus et al. found that aggressive driving style and large road gradients led to increasing CO_2_ emissions, but they had little effect on the CO and HC emissions [23]. In addition, CO_2_, CO, and HC emission rates from gasoline vehicles increased as vehicular speed rose [22,24,25]. However, relatively few studies have been conducted on the CO_2_, CO, and HC emissions under different fuel detergency conditions in gasoline-powered vehicles.

With the increasingly refined requirements of traffic management and policy evaluation, accurate prediction of vehicle transient emissions was becoming more critical [26]. Therefore, the use of gasoline detergents should be considered in model prediction. Traditional models, such as MOVES [27] and IVE [28] which were developed by statistics, could not meet the requirements of this situation. In recent years, machine learning is widely used in the field of motor vehicle emissions prediction. Seo et al. (2021) applied an artificial neural network (ANN) with a vehicle dynamics model to predict the light-duty diesel vehicle’s instantaneous CO_2_ emission [29]. The designed ANN model employed different input variables when predicting various exhaust emission rates. It simultaneously balanced the predictive accuracy and practicality of the model, and utilized cumulative emission values to investigate its predictive precision. Jia et al. (2022) developed a long short-term memory (LSTM)-based microscopic model DL-VEM [30]. The DL-VEM applied an end-to-end structure to predict CO_2_ emissions rate achieving higher accuracy and suitable for both gasoline and compressed natural gas taxicabs. Wei et al. (2022) designed a super-learner model in NOx and CO_2_ emission prediction and explored the most relevant factors of diesel truck exhaust emission [26]. The super-learner model demonstrated significant improvements in both model interpretability and generalization capabilities. In conclusion, the application of machine learning had improved vehicle emission prediction in many aspects, such as higher accuracy and fewer input parameters. To date, no relevant research has considered the influence of detergents in the prediction.

The purpose of this paper was to identify the following problems: (1) characteristics of CO_2_, CO, and HC emissions with different fuel detergency; (2) the synergistic emission reduction potential of CO_2_, CO, and HC under the influence of detergency; (3) the accurate prediction of CO_2_, CO, and HC emission rates considering the different fuel conditions.

## 2. Materials and Methods

### 2.1. Test Vehicle, Routes, and Detergent

In this study, a passenger car (SAIC Volkswagen, Shanghai, China) was selected as the test vehicle which had been one of the best-selling sedans in China for several years. The test vehicle with a model year of 2016 met the China V, which had been driven 59,784 km and was in good working condition. The fuel used in the test was purchased from a local gas station, which met the corresponding Gasoline for motor vehicles (China VI) (GB 17930-2016) [31]. A sufficient quantity of gasoline was prepared and stored in a reserve fuel tank to ensure consistent baseline fuel properties throughout the experimental process.

Zhengzhou is a transportation hub in central China with a huge amount of road passenger and freight traffic. The choice of Zhengzhou as a study area to explore the impact of gasoline detergency is of reference importance to the entire Central Plains region for pollution and carbon reduction studies. To cover the vehicle’s daily working conditions, the test routes, totaling 62.4 km, included urban, suburban, and highway road types. Both experiments were conducted on regular working days to simulate daily driving conditions.

A qualified gasoline detergent [18], which is one of the top-selling products in the Chinese market, was chosen for the real road test. The utilized detergent is a physical additive that can reduce fuel viscosity and surface tension upon application. This enhances fuel atomization during spraying, promoting finer dispersion and improved vaporization, ultimately leading to heightened combustion efficiency. To avoid the influence of gasoline detergent, the first test cycle used gasoline with no detergent added. The effectiveness of the gasoline detergent was influenced by the duration of its usage [32]. To better assess the effect of improved gasoline detergency on emissions, test vehicles used fuel with the detergent continuously for four days before conducting the second round. In the second round of testing, the detergent was added to the same batch of base gasoline following the concentrations recommended in the instructions (0.5–2 v.%). To eliminate the effect of driving habits, two rounds of tests were done by the same driver along the same routes.

### 2.2. Instrumentation and Data Analysis

**Figure 1:**
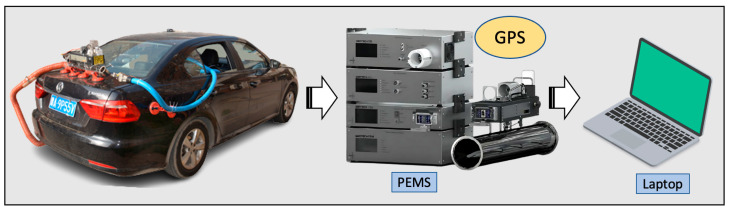
The schematic of the test vehicle and SEMTECH equipment.

In this study, PEMS was used to monitor vehicle emissions data in per second. SEMTECH ECOSTAR PLUS (Sensors Inc., Saline, MI, USA) was chosen as an emissions measurement system because of its high accuracy. The schematic of the test vehicle and SEMTECH equipment are shown in Figure 1.

The per-second average speed *V_i_* in km/h of the test vehicle could be calculated from the GPS second-by-second latitude and longitude data, and the acceleration $a_{i}$ in m/s^2^ was two seconds average of the difference between the previous speed *V_i_*_−1_ and subsequent speed *V_i_*_+1_, as shown in Equation (1).

(1) $$a_{i}=\frac{1}{2}\cdot \left(V_{i+1}-V_{i-1}\right)/3.6$$

Road grade was also close to the vehicle emissions, and it was calculated by the segment method [33]. Depending on the test routes, Δ*d* = 50 m was appropriate for segment length, which was also important for data analysis. Road grade is calculated according to Equation (2):

(2) $$grade=\sum_{i}{\Delta h}_{i}\;\;\;for\;{\Delta h}_{i}>0$$

where Δ*h_i_* was defined by the difference of following from the previous altitude data.

Vehicle specific power (VSP) in kW/ton, first proposed by Jimenez-Palacios in 1998, reflected the current instantaneous output power of the vehicle [34]. Since VSP comprehensively considers vehicle speed, acceleration, and road slope, it can well represent the relationship between vehicle driving conditions and instantaneous emission [35], which is defined as:

(3) $$VSP=V_{i}\cdot \left(1.1\cdot a_{i}+9.81\cdot \alpha +0.132\right)+0.000302V_{i}^{3}$$

where *V_i_* represents the driving speed, $a_{i}$ reflects the acceleration, and *α* means the road grade.

To compare the effects of different route types and gasoline detergent on emissions, the mileage-based emission factor *EF_i_* in g/km was adopted in this study to reflect the impact of different driving conditions on vehicle emissions, as shown in Equation (4).

(4) $${EF}_{i}=\frac{1000\times \sum_{t=j}^{T}{ER}_{i,j}}{\sum_{t=j}^{T}V_{j}}$$

where *EF_i_* in g/km denotes the emission factor of pollution *I*, *T* in s represents the total time consumption of the test cycle, *ER_i_*_,*j*_ in g/s is the emission rate of pollution *i* at second *j*, and *V_j_* in m/s reflects the current driving speed.

### 2.3. The Model of LSTM

**Figure 2:**
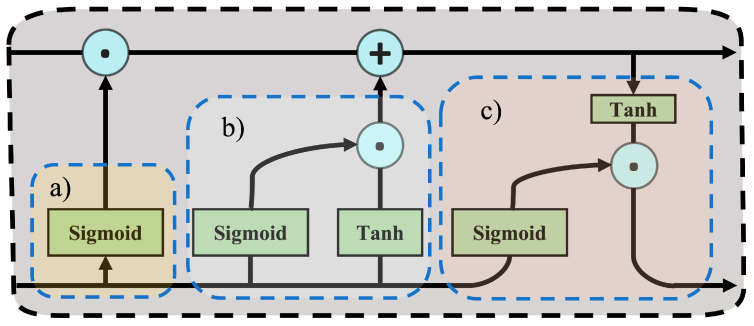
The structure of “LSTM cells”: (**a**) forget gate, (**b**) external input gate, and (**c**) output gate.

Previous studies have concluded that the motor vehicles’ instantaneous exhaust emission rate not only depends on the current driving conditions but also the long-term conditions [26,30,36]. Recurrent neural networks (RNNs) are ones of neural networks that are specialized for processing a sequence of values. LSTM [37] is one of the most effective sequence models named gated RNNs because of having an internal recurrence (a self-loop). As shown in Figure 2, the most important part of LSTM recurrent networks is the “LSTM cells” that include three gated units, namely: forget gate, external input gate, and output gate. The forget gate employs a *sigmoid* function to determine the retention of each past memory unit. The output of the forget gate is constrained between 0 and 1 through *sigmoid* function computation. The value close to 1 signifies the preservation of relevant memories, while proximity to 0 indicates the discarding of pertinent memories. This mechanism empowers the LSTM network to selectively retain the more important information pertinent to the current task, thereby alleviating the issue of vanishing gradients in lengthy sequence data. The external input gate operates by using a *sigmoid* function to determine the degree to which external input information should be integrated. The *sigmoid* function output nearing 1 signifies that the information should be incorporated, whereas an output approaching 0 indicates that the external input information should be attenuated. The output gate, similar to the preceding two gates, utilizes the *sigmoid* function to ascertain the degree to which the information residing within the memory cell should be incorporated into the final output. More detail can be found in our previous study [36].

### 2.4. Model Evaluation

In this study, root-mean-square error (RMSE) was used to measure the difference between observed values and predicted values, and Pearson’s r was adopted to evaluate the correlation between them. The metrics are defined in Equations (5) and (6), respectively.

(5) $$RMSE=\sqrt{\frac{\sum_{i=1}^{N}{\left(\;x_{t}-{\hat{x}}_{t}\right)}^{2}}{N}}$$

(6) $$Pearson’s\;r=\frac{\sum_{t=1}^{N}\left(x_{t}-\bar{x})({\hat{x}}_{t}-{\bar{\hat{x}}}_{t}\right)}{\sqrt{\sum_{t=1}^{N}{\left(x_{t}-\bar{x}\right)}^{2}}\sqrt{\sum_{t=1}^{N}{\left({\hat{x}}_{t}-{\bar{\hat{x}}}_{t}\right)}^{2}}}$$

where $x_{t}$, ${\hat{x}}_{t}$, $\bar{x}$, and ${\bar{\hat{x}}}_{t}$ refer to the *t*-th observed, estimated, actual average, and estimated average of CO_2_, CO, and HC emission rates, respectively.

## 3. Results and Discussion

### 3.1. Exhaust Emissions with Different Gasoline Detergency

#### 3.1.1. Emission Factors on the Various Road Types

**Figure 3:**
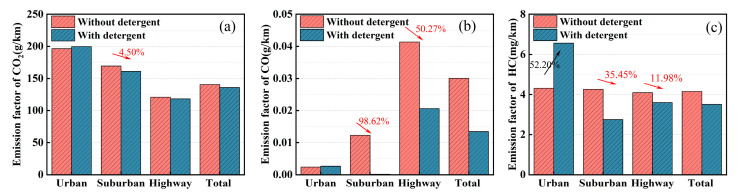
Comparison of exhaust emission factors with and without gasoline detergent on various road types: (**a**) CO_2_, (**b**) CO, and (**c**) HC.

Figure 3 illustrates the effects of road types on the CO_2_, CO, and HC emission factors of the test vehicle under both with and without detergent gasoline conditions. As shown in Figure 3a, the emission factors of CO_2_ from the test vehicles demonstrate consistent changing patterns before and after the introduction of detergent, albeit with disparities in their magnitudes. Additionally, the CO_2_ emission factors follow the order of urban roads > suburban roads > highways. Under non-detergent conditions, the CO_2_ emission factor on highways is 120.79 g/km, representing a reduction of 38.5% compared to urban roads and 28.7% compared to suburban roads. When using detergent, the CO_2_ emission factor on highways decreases to 118.34 g/km, showing a decrease of 40.7% compared to urban roads and 26.5% compared to suburban roads. These findings indicate that different road types exert a significant influence on vehicle CO_2_ emissions, and the utilization of detergents does not alter the fundamental emission characteristics of vehicles.

Under the application of detergent, emissions reduction effects are observed on suburban roads and highways, while a certain degree of emission exacerbation is evident on urban roads. Overall, there is a significant decrease in the total emission factor. Specifically, on suburban roads, the detergency effect is most prominent, resulting in a 5.0% reduction compared to the non-detergent condition. On highways, the CO_2_ emission factor decreases by 2.0% with improved detergent properties. Considering the entire process, the CO_2_ emission factors for the detergent-treated and non-treated conditions are 135.87 g/km and 140.29 g/km, respectively, leading to a 3.2% reduction. Under urban road conditions, the addition of gasoline detergent resulted in a 1.7% increase in the CO_2_ emission factor. Similar to previous research findings, it has been observed that improving gasoline detergency does not yield significant emission reductions during low-speed phases [38]. Another plausible explanation could be the differences in traffic conditions on the testing day. With the detergent added, the vehicle experienced a total of seven start–stop cycles within the urban road, while under non-detergent conditions, there were five start–stop cycles. Frequent start–stop events can lead to emission deterioration [39], potentially contributing to the observed increase in CO_2_ emissions in the experiment with the added detergent.

The relationship between vehicle fuel consumption and its CO_2_ emissions is closely intertwined, and their conclusions often corroborate each other. After calculations, it was determined that the fuel consumption per 100 km after adding detergent was 5.91 L, while it was 6.10 L per 100 km without detergent, resulting in a 5.1% decrease. This reduction can be primarily attributed to carbon deposits in the engine’s intake system and fuel injectors, which affect normal engine intake, increase intake resistance, and reduce combustion efficiency. The utilization of detergent improves the lubrication of the intake system and gasoline and enhances gasoline spray conditions, thereby improving engine performance, reducing fuel consumption, and directly lowering CO_2_ emissions [40].

Figure 3b displays the CO emission factors across different road types. The graph reveals an inverse emission pattern of CO and CO_2_. In the absence of detergent, the CO emission factor is significantly lower on urban roads compared to suburban roads and highways. Specifically, on urban roads, the CO emission factor is 0.0024 g/km, whereas on suburban roads, it increases by over 5 times to 0.01228 g/km, and on highways, it surges by more than 17 times to 0.04136 g/km. This indicates that road types exert a notable influence on CO emission factors, with the hierarchy being highways > suburban roads > urban roads under non-detergent conditions. Upon the application of detergent, the CO emission factor becomes nearly negligible on suburban roads. On urban roads, it remains equivalent to the non-detergent condition at 0.00264 g/km. However, on highways, the emission factor increases, growing by 7.79 times compared to urban roads, reaching 0.02057 g/km. However, compared to the non-detergent condition, the CO emission factor decreases by 50.27%. Overall, improving gasoline detergency demonstrates effective CO emissions reduction on suburban and highway roads. However, its impact on urban roads is not significant, and there is even a slight increase in CO emissions.

Figure 3c illustrates the HC emission factors across different road types and indicates substantial differences before and after the addition of detergent. Before adding detergent, the HC emission factors show minor variations among the three road types, with differences of only 1.4% to 3.9% compared to the total HC emission factor. However, under detergent-treated conditions, the HC emission factors vary significantly among road types. Urban roads exhibit the highest emission factor, reaching up to 6.56 mg/km, corresponding to a 58.1% increase compared to suburban roads and a 36.3% increase compared to highways. In contrast, compared to the non-detergent condition, urban roads experience a substantial increase of 52.2% in HC emissions, while suburban roads show a significant decrease of 35.4%, and highways exhibit a reduction of 12.0%. The overall HC emission factor decreases by 15.4% under detergent-treated conditions. The utilization of detergent leads to a notable increase in HC emissions on urban roads, while it results in significant reductions in HC emissions on suburban and highway roads, as well as the total HC emissions. The emission deterioration on urban roads might be attributed to the initial use of detergent, which disrupts the porous structure of combustion chamber deposits, reducing their adsorption capacity and consequently increasing HC emissions [41]. However, with increasing driving mileage, surface deposits gradually undergo cleaning, leading to a discernible reduction in HC emission factors.

#### 3.1.2. Coupling Effects of Vehicle Speed and Acceleration on Exhaust Emissions

**Figure 4:**
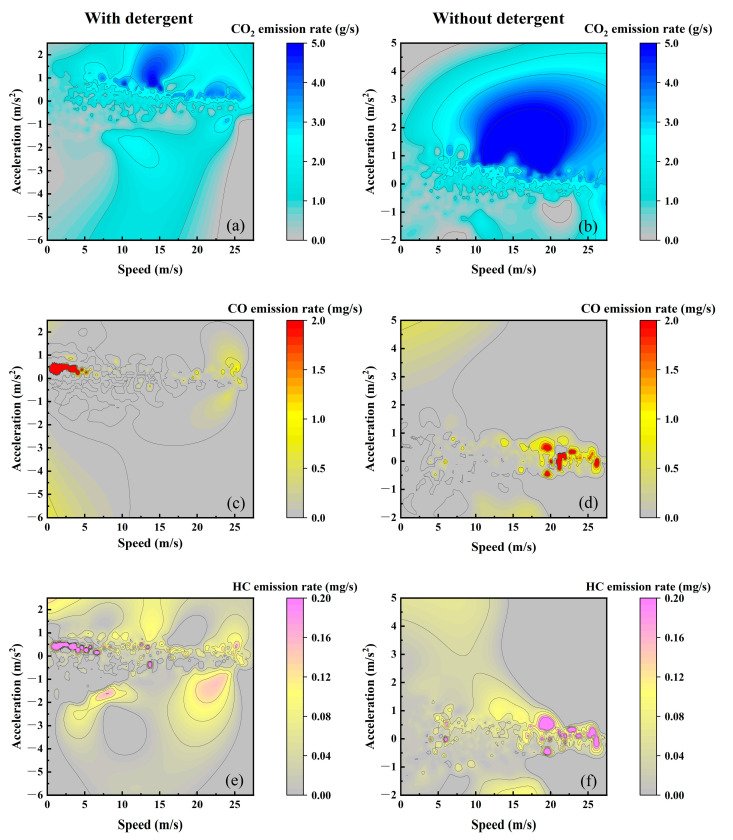
Correlation between vehicle speed, acceleration, and emission rates: (**a**) CO_2_ with detergent; (**b**) CO_2_ without detergent; (**c**) CO with detergent; (**d**) CO without detergent; (**e**) HC with detergent; (**f**) HC without detergent.

Vehicle exhaust emissions are significantly influenced by the operational conditions of the engine, and the vehicle’s driving speed and acceleration have a direct impact on the engine’s performance [22]. Figure 4 illustrates the coupling effects of vehicle speed and acceleration on CO_2_, CO, and HC emission rates of the test vehicle with different detergency fuels under the same test routes. In Figure 4a,b, the graphs illustrate the correlation between vehicle speed, acceleration, and CO_2_ emissions. Figure 4a presents the emissions characteristics without the addition of detergent, while Figure 4b shows the emissions characteristics after utilizing the detergent. From the graph, it is evident that regions with higher CO_2_ emission rates are primarily concentrated during velocity escalation conditions with vehicle speeds > 5 m/s, while CO_2_ emission rates are comparatively lower during deceleration conditions. The application of detergent proves to be effective in ameliorating the CO_2_ emission rates of vehicles under high-speed and velocity escalation conditions. However, for speeds below 15 m/s and acceleration rates less than 1 m/s^2^, the detergent-treated condition exhibits a slight increase in CO_2_ emission rates compared to the non-detergent condition.

In Figure 4c,d, the graphs illustrate the comprehensive relationship between vehicle speed, acceleration, and instantaneous CO emission rates under two different fuel detergency conditions. Figure 4c represents the emission characteristics without the addition of detergent, while Figure 4d depicts the emission characteristics after using the detergent. The graph reveals that under both fuel detergency conditions, CO emissions generally remain at low levels during most of the driving process. However, there are distinctions in the driving states associated with the higher emission rate intervals. Under the non-detergent condition, CO emission rates were relatively higher when the vehicle speed ranged from 16 m/s to 27 m/s, and the acceleration fell within the range of −0.5 m/s^2^ to +0.5 m/s^2^. Conversely, CO emission rates were relatively lower under low-speed conditions. With the addition of the detergent, CO emission rates were comparatively lower during the high-speed driving states. However, when the vehicle speed was in the range of 0 m/s to 5.5 m/s, and the acceleration fell within the range of −0.5 m/s^2^ to +0.5 m/s^2^, CO emission rates were relatively higher.

Figure 4e,f depict the comprehensive relationship between vehicle speed, acceleration, and instantaneous HC emission rates under two different fuel detergency conditions. Specifically, Figure 4e represents the emission characteristics without the addition of detergent, while Figure 4f illustrates the emission characteristics after utilizing the detergent. The graph demonstrates substantial differences in HC emission characteristics under the two fuel detergency conditions. Interestingly, the higher emission rate intervals for HC closely resemble those observed for CO emissions. Under the non-detergent condition, higher HC emission rates are observed when the vehicle speed is >17 m/s, and the acceleration falls within the range of −0.7 m/s^2^ to +0.8 m/s^2^. Occasionally, there are some instances of abnormal emissions near the vehicle speed of 6 m/s, while HC emission rates remain relatively low under other conditions. Upon adding the detergent, the higher emission areas are mainly concentrated when the vehicle speed is below 8 m/s and the acceleration is within the range of 0 m/s^2^ to 0.8 m/s^2^. Additionally, sporadic higher emissions are observed when the vehicle speed is near 15 m/s. Under other conditions, the HC emission rates remain relatively low.

#### 3.1.3. Exhaust Emissions Distribution in Accordance with Vehicle VSP

**Figure 5:**
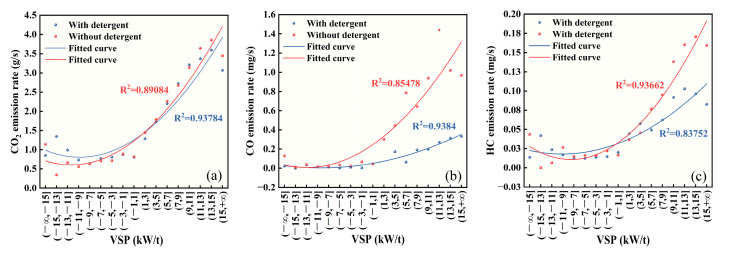
Relationship between emission rates and VSP under different fuel detergency conditions: (**a**) VSP-CO_2_, (**b**) VSP-CO, and (**c**) VSP-HC.

VSP refers to the actual power output of a vehicle during its operation, providing a comprehensive representation that incorporates vehicle speed, acceleration, and road gradient. Prior research has shown a strong correlation between VSP and automotive pollutant emissions, as well as CO_2_ emissions [42]. Figure 5 shows the relationship between VSP and CO_2_, CO, and HC emission rates under different gasoline detergency conditions. Figure 5a illustrates the variations in CO_2_ emissions with respect to VSP for the test vehicle before and after adding detergent. The horizontal axis represents VSP Bin intervals, while the vertical axis denotes the average CO_2_ emission rate within each interval. The graph reveals that the fundamental characteristics of CO_2_ distribution with VSP remain consistent under both fuel detergency conditions. This is attributed to the fact that as the actual output power of the vehicle rises, fuel consumption also increases, leading to a subsequent rise in CO_2_ emissions. When VSP < 0, the CO_2_ emission rates for both conditions are relatively small, generally below 1.0 g/s. Conversely, when VSP > 0, the CO_2_ emission rates increase sharply with the rise in VSP. Moreover, when VSP < −11, both fuel detergency conditions exhibit a slight upward trend in CO_2_ emission rates, which corresponds to the vehicle’s rapid deceleration conditions. From the data analysis in Figure 5a, it is evident that the application of detergent significantly reduces CO_2_ emission rates in the VSP range greater than 11 and lower than −15. However, it appears to have a slight exacerbating effect on emissions in the range from −15 to −9. In the remaining VSP ranges, CO_2_ emission rates remain comparable between both fuel detergency conditions. The fitted curves also demonstrate that detergent usage effectively lowers CO_2_ emission rates in the larger VSP ranges, while increasing them in the smaller VSP ranges.

In Figure 5b, the distribution of CO emission rate with respect to VSP for the test vehicle before and after adding detergent is depicted. The horizontal axis represents VSP Bin intervals, while the vertical axis displays the average CO emission rate within each interval. The results show notable disparities in the CO emission patterns under different fuel detergency conditions. Under non-detergent conditions, the fitted curve reveals that CO emission rates exhibit a significant increasing trend when VSP values exceed −7, while they experience only minor variations at lower VSP values (below −7). Upon adding detergent, a noticeable increase in CO emission rates with rising VSP values are observed after VSP exceeds 3, although the rate of increase is significantly lower compared to the non-detergent condition. Overall, there exists a critical point where the CO emission rate gradually starts to increase with higher VSP values. The utilization of detergent extends the range of VSP values with lower CO emissions, providing more driving states with reduced CO emission levels. Furthermore, the data distribution and curve-fitting analysis reveal that enhancing gasoline detergency effectively reduces CO emissions when VSP is positive, resulting in a nearly 80% decrease in the maximum emission rate compared to the non-detergent condition.

Figure 5c is the distribution of HC emission rates with respect to VSP for the test vehicle before and after adding gasoline detergent. The *x*-axis represents VSP Bin intervals, while the *y*-axis displays the average HC emission rates within the corresponding intervals. It is evident that when VSP is less than 1, HC emission rates remain relatively low for both conditions. Additionally, when VSP is less than −9, both conditions exhibit slight variations in HC emission rates. On the other hand, for VSP values exceeding 1, the average HC emission rates start to rise significantly, with the rate of increase being lower after the introduction of the detergent compared to the pre-additive phase. Moreover, a similar trend is observed for higher VSP values in both conditions: before detergent addition, the HC emission rates reach their peak when VSP values are within the range of 13 to 15, followed by a gradual decline. After detergent addition, the peak emission rate is observed when VSP values are in the range of 11 to 13, subsequently leading to a decline in emission rates. Overall, the utilization of detergent results in an advanced peak of HC emission rates with increasing VSP values and demonstrates effective reduction of HC emissions at higher VSP levels. However, its influence on HC emissions at lower VSP levels remains relatively consistent.

### 3.2. Synergistic Emission Reduction Potential of Enhanced Detergency

#### 3.2.1. The Pollutant Emission Indicator

In this subsection, the potential of gasoline detergency to synergistically reduce CO_2_, CO, and HC emissions in gasoline vehicles is further investigated. The emission indicator (EI), which represents the ratio of pollutant mass (or quantity) to CO_2_ mass, is employed to analyze the emission behavior of various pollutants from vehicles. Typically, pollutant emission indices are based on fuel consumption, and since CO_2_ emissions are directly related to fuel consumption, this study calculates the pollutant mass emitted per kilogram of CO_2_. As the emission indicator comprehensively considers both CO_2_ and gaseous pollutant emissions, it also reflects the synergistic emission reduction efficiency of CO or HC under conditions of equal CO_2_ emissions [43]. Moreover, previous research has shown that the emission index analysis can mitigate the impact of factors such as cold starts on emissions [44]. The calculation formulas adopted in this study are as follows:

(7) $$EI=\frac{1000M}{M_{{CO}_{2}}}$$

where *EI* in g/kg denotes the emission indicator, *M* in g represents the mass of CO or HC, and $M_{{CO}_{2}}$ in g reflects the mass of CO_2_.

#### 3.2.2. The Distribution of Pollutant Emission Indicators Based on VSP

**Figure 6:**
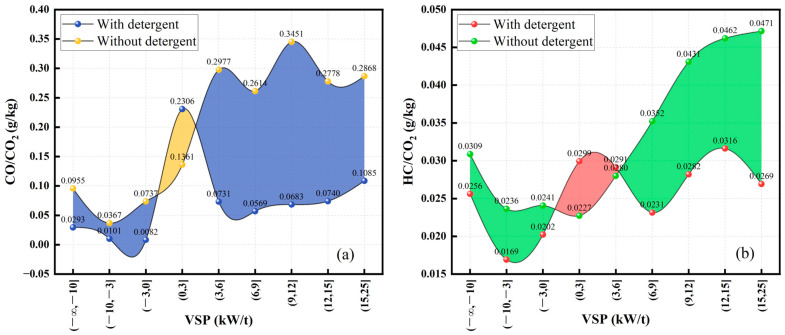
Emission indicator distribution with VSP: (**a**) CO/CO_2_ and (**b**) HC/CO_2_.

As shown in Figure 6, the analysis focuses on the distribution of emission indications with respect to VSP to comprehensively assess the collective influence of speed, acceleration, and road gradient. In Figure 6a, the CO emission indicator is analyzed with respect to VSP Bin. The yellow dots represent the CO/CO_2_ ratio without the addition of detergent, while the blue dots represent the ratio after the addition. The yellow region indicates the reduction in CO emissions per 1 kg of CO_2_ emitted due to the addition of detergent, while the blue region represents the additional CO emissions emitted. From Figure 6a, it is evident that the majority of VSP intervals show a reduced CO/CO_2_ ratio after the application of detergent, indicating a reduction in the amount of CO produced per unit of fuel consumption. However, a slight increase in the CO emission indicator is observed when VSP ranges from −2 to 4. The analysis of area proportions reveals that the additional CO emissions per 1 kg of CO_2_ emitted after detergent addition are significantly lower than the achieved reduction. Thus, enhancing gasoline detergency proves to be an effective approach for achieving synergistic CO and CO_2_ emission reductions throughout the complete vehicle driving cycle.

Figure 6b reflects the distribution of HC emission indicator with respect to VSP Bin. The red data points represent the HC/CO_2_ ratio after using the detergent, while the green data points represent the ratio without. The green region corresponds to the emission reduction effect achieved after using the detergent, while the red region represents the emission exacerbation scenario. The utilization of detergent demonstrates favorable emission reduction effects in the majority of VSP ranges, while a deterioration in emissions is observed when VSP falls within the range of −2 to 6. This observation aligns with the CO emissions, showcasing a similar pattern of cooperative reduction. However, the graph indicates that the CO emissions demonstrate a more pronounced synergistic reduction effect in comparison to HC emissions. Tolga’s study also yielded similar findings, demonstrating that the use of fuel additives improved CO and HC emissions. However, it was observed that the reduction in HC emissions was not as significant as the reduction in CO emissions [45].

In general, the utilization of detergent fuel leads to a significant synergistic reduction effect on CO_2_, CO, and HC emissions. The reduction effect on CO emissions surpasses that of HC emissions. However, it is noteworthy that both CO and HC emissions exhibit a slight deterioration near the VSP value of 0.

### 3.3. Emission Models of Gasoline Vehicles under Different Detergency Conditions

#### 3.3.1. Modelling Feature Analysis

**Figure 7:**
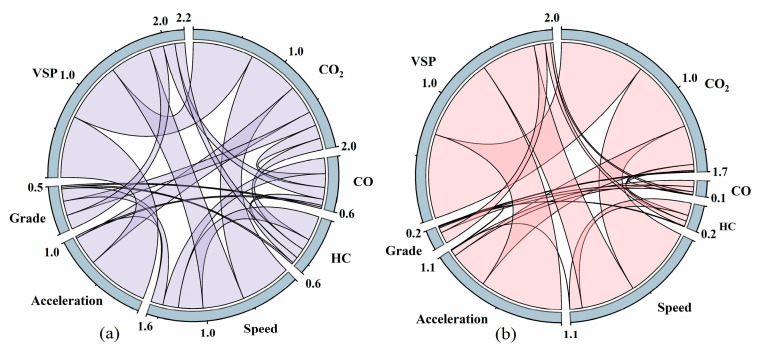
Correlation between model input features and predicted values: (**a**) without detergent, (**b**) with detergent.

Pearson’s r was used to analyze the correlation between the model’s input features and the predicted values in this subsection. Figure 7 illustrates the magnitudes of Pearson’s r between different variables. Each of the seven partitions represents an involved variable, and the wider lines connecting the two variables indicate a higher correlation between them.

As shown in Figure 7a,b, vehicle speed acceleration and VSP exhibit the most evident linear correlations with CO_2_ emissions. This is attributed to the fact that gasoline detergents do not modify the fundamental emission characteristics, and the rise in vehicle output power, acceleration, and high-speed operation leads to an increase in CO_2_ emissions. After the addition of the gasoline detergent, the correlation between CO_2_ and HC, as well as CO, decreases. Similarly, the correlations between HC and CO with other variables also decrease. This observation can be attributed to the significant reduction in HC and CO emission rates during the medium-to-high-speed stages after the addition of the detergent. Consequently, the lower emission values result in decreased correlations with other variables.

#### 3.3.2. Data Preparation and Model Training

Algorithm 1 demonstrates the process of constructing the training dataset for the model. The input data included the vehicle driving conditions data (*X*), comprising speed and acceleration, historical emission data (*Y*) for CO_2_, CO, and HC, and external influencing data (*Z*) including road grade. An empty set was established to store the training data, then formatted training data were added to it. In order to improve the model’s computational efficiency, the training data were normalized to the range of (0, 1).
**Algorithm 1.** Feature organization.1: Initialize a train dataset $D_{train}\leftarrow \emptyset$
2:   **For** *t* = *p* to *n*3:       Input data $X=\left\{x_{t-p+1},x_{t-p+2},\cdots ,x_{t-1},x_{t}\right\};$4:           $Y_{his,i}=\left\{y_{t-p+1},y_{t-p+2},\cdots ,y_{t-1}\right\},Y_{t,i};$5:           $Z=\left\{z_{t-p+1},z_{t-p+2},\cdots ,z_{t-1},x_{t}\right\};$6:           $Q=\left[X,Y_{his,i},Z\right];$7:          Insert (*Q*, $Y_{t,i}$) into $D_{train};$8:   **End**

Where $D_{train}$ represents the training dataset, *p* is the input sequence length, *X* denotes the historical and current vehicle driving data, *Y_his,i_* mean the historical exhaust emission data, *Y_t,i_* is the current emission data, *Z* denotes the historical and current external influencing data, and *Q* represents organized training data.

This study was conducted using PyCharm as the integrated development environment for Python 3.8. The backend utilized was TensorFlow 2.4.1, and the emission model framework was constructed using Keras. The experiments were performed on the macOS operating system equipped with an Intel Core i5-8279U 1.4 GHz processor and 8 GB of memory. Algorithm 2 demonstrates the model training process. Initially, the model parameters were initialized randomly, and the overall loss was initialized to 0. The forward propagation was performed multiple times to compute predicted values, while the backward propagation was used to optimize the model parameters until the model loss value reached the specified accuracy threshold. Eventually, the trained DPVEM-DGD was exported.
**Algorithm 2.** Model training.1:   Initialize model parameters randomly as Θ, and total loss as *Loss* = 0;2:   **Repeat**3:      **For each**
${(Q,Y}_{t,i})$ **in** $D_{train}$4:         Perform forward propagation to compute $\hat{Y_{t,i}}$;5:         Compute mean square error $L\left(\Theta \right)={\left\|Y_{t,i}-\hat{Y_{t,i}}\right\|}^{2}$;6:         $Loss+=L\left(\Theta \right)$;7:         Perform backward propagation to compute∆Θ; 8:         update Θ, Θ=Θ+∆Θ;9:      **End**10:   **Until** Loss<σ;11:   Output: Trained model DPVEM-DGD

Where Θ is the model parameters, *Loss* represents the loss value of the model, $\hat{Y_{t,i}}$ denotes the predicted values of different exhaust emissions, $L\left(\Theta \right)$ is the result of the loss function, ∆Θ represents the change in parameters, and σ is the specified accuracy threshold.

#### 3.3.3. Model Validation and Discussion

**Figure 8:**
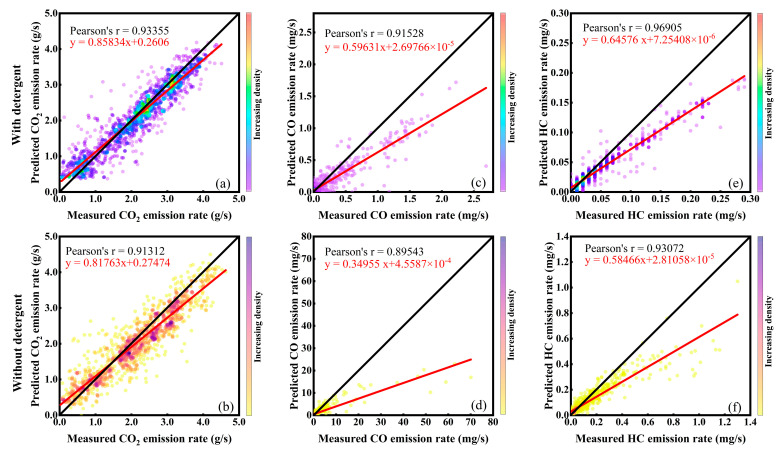
Emission estimation results of DPVEM-DGD model: (**a**) CO_2_ with detergent; (**b**) CO_2_ without detergent; (**c**) CO with detergent; (**d**) CO without detergent; (**e**) HC with detergent; (**f**) HC without detergent.

Pearson’s r was utilized to reflect the linear regression relationship between the measured values and the DPVEM-DGD model predicted values. MSE was used to evaluate the accuracy of the model by computing the average squared difference between the predicted values and the measured values. Under the non-addition of detergent condition, Pearson’s r values of CO_2_, CO, and HC were 0.91312, 0.89543, and 0.93072, respectively. The MSE values of CO_2_, CO, and HC were 0.20, 1.14 × 10^−5^, and 6.20 × 10^−9^, respectively. After the introduction of detergent, Pearson’s r values of CO_2_, CO, and HC were 0.93355, 0.91528, and 0.96905, respectively. The MSE values of CO_2_, CO, and HC were 0.14, 2.52 × 10^−8^, and 4.45 × 10^−10^, respectively. Pearson’s r was close to 1 for both detergency conditions, indicating a strong linear relationship between the predicted and measured values. Additionally, the small MSE values suggest that the model’s predictions have minimal error compared to the actual measurements. These findings demonstrate the DPVEM-DGD model’s excellent performance in predicting exhaust emissions, which could provide accurate predictions for both conditions. After the addition of detergent, the DPVEM-DGD model exhibited improved performance in predicting CO_2_, CO, and HC, as evident from the higher Pearson’s r values and lower MSE values compared to the pre-addition scenario [26]. Moreover, the fitted regression lines were even closer to the perfect line (*y = x*), as illustrated in Figure 8. The results indicated that improving the detergency of the gasoline could effectively enhance the predictive performance of the model for instantaneous exhaust emission rates. This improvement was likely attributed to the enhanced gasoline detergency, which led to improved engine combustion and reduced peak emissions during sharp increases, resulting in smoother emission rates. Consequently, the prediction difficulty was reduced, resulting in better predictive performance.

**Figure 9:**
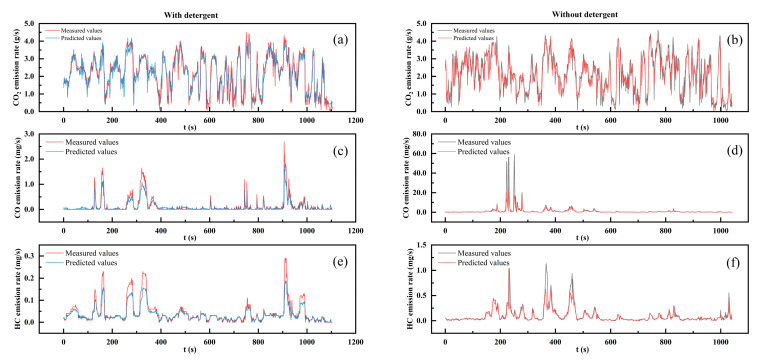
Validation of DPVEM-DGD model–predicted results with measured data: (**a**) CO_2_ with detergent; (**b**) CO_2_ without detergent; (**c**) CO with detergent; (**d**) CO without detergent; (**e**) HC with detergent; (**f**) HC without detergent.

To validate the predictive accuracy of the DPVEM-DGD model, a comparison was conducted between the predicted values and measured values, as shown in Figure 9. From the graph, it was evident that the model could effectively predict the instantaneous emission rates of different exhaust gases under both detergency conditions. The results of all six times predictions indicated that the model was capable of forecasting the occurrence of emission peaks. However, there existed a certain discrepancy between the predicted values of peak magnitudes and the measured values. The potential cause for the limited accuracy in peak value predictions could be attributed to the relatively limited size of the training dataset, resulting in inadequate learning of the peak emission characteristics by the model [29]. Another potential factor could contribute to the influence of various external factors. Achieving precise peak predictions could require incorporating a broader range of input features for the prediction process [46]. Upon comparative analysis of Figure 9c–f, it was evident that the introduction of the detergent had resulted in a mitigated increase of peak values in the instantaneous emission rates of CO and HC. This amelioration positively influenced the accuracy of predicting their peak values. As shown in Figure 9c,e, the instantaneous emission rates of HC and CO exhibited similar changing patterns with a simultaneous increase after the addition of detergent, whereas no such correlation was observed before the addition of detergent. One possible conjecture was that the addition of detergent clears partial carbon deposits in the engine’s intake system, reducing the intake pressure of the engine and thereby mitigating the influence of unstable external factors. This may lead to a more apparent emission pattern, increasing the likelihood of achieving accurate predictions.

**Figure 10:**
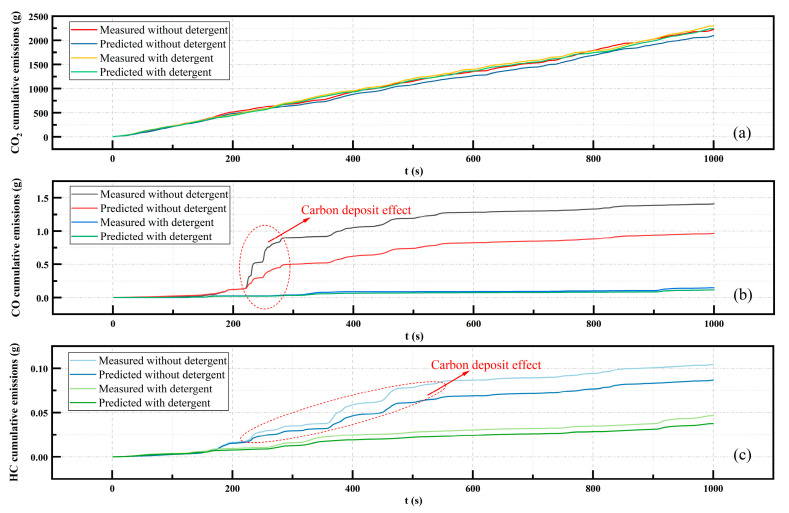
Impact of detergent addition on exhaust gas accumulation and predictions: (**a**) CO_2_, (**b**) CO, and (**c**) HC.

As shown in Figure 9, the emissions of CO and HC from vehicles were low in most cases, but there were certain regions where the emissions showed a significant increase. Figure 10 presents the cumulative emissions of the exhaust measured values and predicted values taken from the test dataset for a continuous period of 1000 s. As shown in Figure 10a, the cumulative emissions predictions of CO_2_ under both conditions were relatively close to the measured values, but the difference between the predicted and measured cumulative emissions was smaller in the additive condition.

From Figure 10b,c, it was evident that the detergent’s impact on the cumulative emissions and predictions of CO and HC was substantial. Under the non-addition condition, the sudden increase of CO and HC emissions contributed significantly to the total emissions, which was consistent with findings from previous studies [29,47]. The inaccurate prediction of sudden increases in emissions also resulted in significant deviations in the total cumulative emissions predictions. However, after a period of using the detergent, the suppression of sudden emission increases led to smaller cumulative emissions for CO and HC. The smaller discrepancy between the predicted and measured values after adding the detergent also led to a more accurate prediction of cumulative emissions. This indicated that improving gasoline detergency contributed to the enhanced predictive accuracy of exhaust emissions during the gasoline vehicle’s real-world driving. Such improvement could be positive for the more precise management and control of vehicle emissions.

## 4. Conclusions

To evaluate the gasoline detergency in reducing carbon and pollution emissions, this study employed PEMS for real driving tests, quantitatively exploring the emissions characteristics of CO_2_, CO, and HC. DPVEM-DGD was developed for different fuel detergency conditions. The primary findings are as follows:

Firstly, emission rates of CO_2_, CO, and HC correlate with variables such as speed, acceleration, VSP, and road types. CO_2_ emissions exhibit similar patterns under different fuel detergency. Improving fuel detergency reduces emission rates of higher speeds, accelerations, and VSP conditions. However, at lower speeds and smaller VSP, emissions slight increase. Detergency greatly impacts CO emissions reduction, with noticeable reductions except at low speed and near-zero VSP. The reduction in HC emissions is slightly less pronounced compared to CO, yet it still exhibits a considerable emission reduction effect. The results indicate that enhancing fuel detergency can effectively achieve synergistic emission reduction while simultaneously reducing CO and HC emissions.

Secondly, the DPVEM-DGD model can accurately predict CO_2_, CO, and HC emission rates under various fuel detergency conditions. Additionally, improved fuel detergency results in smoother variations in exhaust emission rates, thereby reducing the complexity of precise prediction. This enhancement in fuel detergency further enhances the accuracy of predicting instantaneous emission rates and facilitates more effective management of exhaust emissions.

Moreover, study limitations stem from singular experimentation on a commonly owned vehicle. Whether similar emission characteristics exist for vehicles of varying model years, mileage, and emission standards requires further investigation for confirmation.

## Data Availability

The data that support the findings of this study are in this paper.
